# Draft Genome Sequence of a Multidrug-Resistant *Klebsiella pneumoniae* Carbapenemase-Producing *Acinetobacter baumannii* Sequence Type 2 Isolate from Puerto Rico

**DOI:** 10.1128/genomeA.00758-16

**Published:** 2016-08-18

**Authors:** Teresa Martínez, Alexander J. Ropelewski, Ricardo González-Mendez, Guillermo J. Vázquez, Iraida E. Robledo

**Affiliations:** a Department of Microbiology and Medical Zoology, University of Puerto Rico, School of Medicine, San Juan, Puerto Rico; b Pittsburgh Supercomputing Center, Carnegie Mellon University, Pittsburgh, Pennsylvania, USA; c Department of Radiological Sciences, University of Puerto Rico, School of Medicine, San Juan, Puerto Rico

## Abstract

We report here the draft genome sequence of *Acinetobacter baumannii* strain M3AC14-8, sequence type 2 (ST2), carrying a chromosomally carried *bla*_KPC-2_ gene. The draft genome consists of a total length of 4.11 Mbp and a G+C content of 39.25%.

## GENOME ANNOUNCEMENT

*Acinetobacter baumannii* is associated with severe infections that have high morbidity and mortality. Treatment of infections caused by this organism is hampered by the emergence of antibiotic-resistant isolates. The mechanisms of low-level resistance to carbapenems include reduced antibiotic uptake due to loss of porins combined with derepression of the chromosomal AmpC β-lactamase gene and/or the overexpression of an efflux pump system (1). β-Lactamases are the most common mechanism of resistance to the β-lactam antibiotic. They are frequently located on mobile genetic elements, which facilitates their inter- and intraspecies dissemination. Carbapenems are commonly the last-resort antibiotics for the treatment of infections caused by multidrug-resistant Gram-negative bacilli. The *Klebsiella pneumoniae* carbapenemase (KPC) enzyme hydrolyzes all known β-lactam antibiotics, including the carbapenems (2).

The KPC-producing *A. baumannii* strain M3AC14-8 was isolated from the skin of a 62-year-old female patient hospitalized in the intensive care unit. The strain belongs to the globally disseminated sequence type 2 (ST2). Next-generation sequencing was performed using an Illumina MiSeq platform in a 2 × 150-bp paired-end (PE) configuration by Genewiz, Inc. The Pittsburgh Supercomputing Center Blacklight supercomputer system (3) was used to performed *de novo* assembly and preliminary genome annotation. *De novo* assembly was done using a combination of Velvet (version 1.2.10) (4), with additional scaffolding provided through SSPACE Basic 2.0 (5). Additional PCR and sequencing were performed to join the resulting scaffolds. The draft genome of *A. baumannii* strain M3AC14-8 consists of 30 scaffolds, with a total length of 4,117,354 bp, an average contig length of 146,869 bp, a maximum contig length of 521,258 bp, and two complete and circularized plasmids of 5,441 and 18,043 bp. The G+C content was determined to be 39.25%.

Genome annotation was performed using the National Center for Biotechnology Information (NCBI) Prokaryotic Genome Annotation Pipeline and was corroborated using the complete proteome of *A. baumannii* strain MDR-ZJ06 (accession no. CP001937), and 3,746 ORFs with *E* values of ≤1e-5 were common to the two genomes. The Pfam database gave 2,285 unique protein families (6). A total of 65 tRNA genes and nine rRNA were identified using tRNAscan-SE (7) and RNAmmer (8), respectively.

In addition to the chromosomally carried KPC-2 gene, ResFinder-2.1 at the Center for Genomic Epidemiology server (9) identified the presence of multiple resistance genes against aminoglycoside, β-lactam, fluoroquinolone, macrolide, chloramphenicol, sulfonamide, and tetracycline antibiotics in the genome. No resistance genes were associated with the two plasmids identified. Further analysis identified the presence of AdeIJK and AdeABC efflux pumps with amino acid mutations in the regulatory genes (*adeRS*) as well as amino acid mutations within the *gyrA* and *parC* genes.

### Accession number(s).

This whole-genome shotgun project has been deposited at DDBJ/ENA/GenBank under the accession no. LDDY00000000. The version described in this paper is version LDDY01000000.
